# Differential Effects of Donepezil and Tacrine on Recall-Phase Exploration in a Trihexyphenidyl-Induced Cholinergic Impairment Y-Maze Model

**DOI:** 10.3390/biomedicines14040938

**Published:** 2026-04-20

**Authors:** Adrian-Florentin Dragomir, Smaranda Stoleru, Aurelian Zugravu, Elena Poenaru, Maria Carina Dumitrescu, Aurelia Cristiana Barbu, Silvia Fratea, Clara Maria Stoleru, Oana Andreia Coman, Ion Fulga

**Affiliations:** 1 Faculty of Medicine, “Carol Davila” University of Medicine and Pharmacy, 050474 Bucharest, Romania; adrian-florentin.dragomir@drd.umfcd.ro (A.-F.D.); smaranda.stoleru@umfcd.ro (S.S.); maria-carina.dumitrescu@rez.umfcd.ro (M.C.D.); aurelia-cristiana.barbu@drd.umfcd.ro (A.C.B.); oana.coman@umfcd.ro (O.A.C.); ion.fulga@umfcd.ro (I.F.); 2 Service Anesthesie-Reanimation, Groupe Hospitalier de la Pitié-Salpêtrière, 75651 Paris CEDEX 13, France; silvia.fratea@aphp.fr; 3 Faculty of Medical Engineering, National University of Science and Technology Politehnica Bucharest, 060042 Bucharest, Romania; clara_maria.stoleru@stud.fim.upb.ro

**Keywords:** trihexyphenidyl, donepezil, tacrine, Y-maze, cholinergic impairment, recall-phase exploration, acetylcholinesterase inhibitors, butyrylcholinesterase

## Abstract

**Background/Objectives:** Cholinergic dysfunction plays a central role in memory impairment, yet trihexyphenidyl (THP)-based paradigms remain less explored than scopolamine-based models. This study aimed to characterize a THP-induced cholinergic challenge in a two-trial Y-maze with a 24 h interval and to compare the effects of donepezil and tacrine on recall-phase exploratory allocation. **Methods:** Male Wistar rats (*n* = 9/group) were studied in a validation phase including saline, THP 5 mg/kg, and THP 10 mg/kg groups, followed by an intervention phase including control, THP 10 mg/kg, donepezil 1 and 3 mg/kg + THP, and tacrine 3 and 5 mg/kg + THP groups. All treatments were administered intraperitoneally (i.p.). Acquisition- and recall-phase behavior was analyzed. Recall outcomes included arm times, arm entries, the novel-to-familiar arm time ratio (U/K time ratio), the novel-to-familiar arm entry ratio (U/K entry ratio), discrimination indices and time-per-entry indices. Data were analyzed by one-way ANOVA; Tukey’s post hoc test was used in the validation experiment, whereas Dunnett’s test was used in the intervention experiment for comparisons against THP 10. **Results:** THP at 10 mg/kg produced a robust recall-phase phenotype, with increased familiar-arm exploration, reduced novel-arm exploration and lower normalized indices. Under THP challenge, donepezil was associated with clearer effects at 3 mg/kg, whereas tacrine displayed a broader dose-dependent profile, with the strongest shift in recall-phase exploratory allocation toward the novel arm observed at 5 mg/kg. **Conclusions:** THP 10 mg/kg produced a robust recall-phase exploratory phenotype in a 24 h two-trial Y-maze paradigm. Under THP challenge, donepezil and tacrine were associated with shifts in recall-phase exploratory allocation. These findings support the potential utility of THP-based paradigms for studying cholinergic disruption in Y-maze settings, while direct comparison with scopolamine-based models remains to be established.

## 1. Introduction

Alzheimer’s disease (AD) is a progressive neurodegenerative disorder and the leading cause of dementia, characterized clinically by a gradual decline in memory and other cognitive domains that can ultimately compromise independent daily functioning [1,2]. Although diagnostic frameworks have evolved alongside with the growing availability of biomarkers that allow earlier biological characterization, current pharmacological options remain largely symptomatic and do not slow long-term disease progression [3,4].

Disruption of muscarinic receptor signaling has been closely associated with the cognitive deficits observed in Alzheimer’s disease [5]. Degeneration of basal forebrain cholinergic neurons reduces acetylcholine release in cortical and hippocampal circuits, thereby diminishing muscarinic receptor activation and compromising synaptic communication within networks supporting learning and memory [5,6]. In particular, reduced M1 receptor-mediated signaling has been linked to learning- and memory-related behaviors in preclinical settings [7].

Animal models are essential tools for investigating the neurobiological mechanisms underlying cognitive impairment and for evaluating potential therapeutic agents [8,9]. Because the full pathological complexity of Alzheimer’s disease is difficult to reproduce experimentally, a range of experimental models—including pharmacological challenge paradigms—have been developed to capture specific neurochemical and behavioral components of the disorder [8,9].

Among pharmacological approaches, centrally acting antimuscarinic agents provide a practical method to induce transient cholinergic disruption and to probe muscarinic contributions to cognition-related performance. Trihexyphenidyl (THP) is a centrally acting antimuscarinic agent with preferential affinity for M1 muscarinic receptors commonly prescribed for Parkinson’s disease and drug-induced extrapyramidal symptoms [10,11].

The cognitive impact of anticholinergic drugs has attracted increasing attention, as accumulating evidence suggests that a high anticholinergic burden may contribute to cognitive impairment and increase dementia risk [12]. Consistent with this, experimental studies have shown that anticholinergic compounds—including scopolamine, trihexyphenidyl, and related agents—can impair multiple cognitive domains, such as attention, immediate recall, and recognition memory [13,14,15]. Together, these observations underscore the importance of intact cholinergic signaling for cognitive function and motivate careful evaluation of drugs that interfere with muscarinic receptor activity.

In preclinical research, acute administration of THP has been employed as a pharmacological challenge to induce transient cholinergic disruption and to probe muscarinic contributions to learning- and memory-related performance (e.g., passive avoidance or other mnemonic tasks) [16,17]. Chronic exposure has been associated with broader neurobiological changes, including neuroinflammation, microgliosis, tau misfolding, and altered neuroimmune responses in aging rodents [18].

Compounds that enhance cholinergic neurotransmission have been extensively investigated for their ability to attenuate antimuscarinic-induced cognitive deficits in animal models, most commonly using scopolamine-based pharmacological challenges [19,20]. In contrast, trihexyphenidyl-induced models remain comparatively underexplored, although they may offer a useful pharmacological approach for examining muscarinic-related cognitive dysfunction [19,21].

Among the cholinergic enhancers, acetylcholinesterase (AChE) inhibitors represent a major symptomatic strategy as they increase acetylcholine availability at cholinergic synapses and can partially offset the consequences of muscarinic blockade [22]. In preclinical studies, cholinesterase inhibitors have been shown to improve performance in various behavioral domains, including spatial learning, passive avoidance, and recognition memory tasks, thereby supporting the functional importance of cholinergic tone in memory processes [20,22].

Tacrine was the first centrally acting cholinesterase inhibitor approved for the symptomatic treatment of Alzheimer’s disease [23]. It is a reversible, non-selective inhibitor of both AChE and butyrylcholinesterase (BuChE), and it remains an important reference compound in experimental pharmacology despite reduced clinical use due to hepatotoxicity [24,25]. Donepezil is a reversible AChE inhibitor with relatively greater selectivity for AChE and has also been widely evaluated in pharmacological impairment models [22,26]. Notably, the distinct enzymatic profiles of tacrine (dual AChE/BuChE inhibition) versus donepezil (predominantly AChE inhibition) raise the possibility of different behavioral signatures under antimuscarinic challenge conditions, including differential effects on recall-phase exploratory allocation as measured in Y-maze paradigms.

In the present study, we first validated an acute THP-based cholinergic challenge in a two-trial Y-maze paradigm with a 24 h interval by comparing THP doses to identify the most robust recall-phase phenotype. We then examined, in THP-challenged animals, whether selective AChE inhibition (donepezil) versus dual AChE/BuChE inhibition (tacrine) differentially modulates recall-phase exploratory allocation. Outcomes included time spent in the familiar and novel arms, normalized novel-to-familiar arm ratios (U/K ratios), discrimination indices for time and entries, entry-based measures and their ratios, and time-per-entry indices, enabling comparison of arm-selection dynamics versus changes in visit duration during the recall session.

## 2. Materials and Methods

The study protocol was approved by the Ethics Committee of the “Carol Davila” University of Medicine and Pharmacy, Bucharest, Romania (approval no. 13619/24.05.2024). All experimental procedures were performed in accordance with national legislation regarding the protection of animals used for scientific purposes and under the supervision of the National Sanitary Veterinary and Food Safety Authority (approval no. 9/05.07.2024).

### 2.1. Animals

Male Wistar rats, aged 6–8 weeks, weighing 200–300 g at testing, were provided by the bio-base (animal breeding unit) of “Carol Davila” University of Medicine and Pharmacy, Bucharest, Romania. The animals were acclimatized to laboratory conditions for at least 7 days before the start of the experiments and were housed individually under standard laboratory conditions with free access to food and water (ad libitum), natural ventilation, ambient humidity maintained between 40% and 60% and a natural light cycle consistent with institutional housing conditions. Individual housing was used to prevent social interaction-related variability in exploratory behavior.

The study was conducted in accordance with the ARRIVE guidelines for reporting animal research.

All experiments were conducted in the Department of Pharmacology between 10:00 and 17:00. Each animal was included in only one experimental protocol. Twenty-four hours after completion of behavioral testing, the animals were euthanized under general anesthesia.

### 2.2. Drugs and Reagents

The following substances were used as standardized preparations:

- Trihexyphenidyl hydrochloride (THP) (500 mg vials; Manufacturer: MedChemExpress, Monmouth Junction, NJ, USA; Product code: HY-B1277).
- Donepezil hydrochloride (1 g vials; Manufacturer: Sigma-Aldrich/Merck, St. Louis, MO, USA; Product code: D6821; purity: ≥98% by HPLC).
- Tacrine [9-amino-1,2,3,4-tetrahydroacridine hydrochloride hydrate] (1 g vials; Manufacturer: Sigma-Aldrich/Merck, St. Louis, MO, USA; Product code: A79922; Purity: ≥99%).
- 0.9% sodium chloride solution (500 mL; Manufacturer: B.Braun, Melsungen, Germany).

All substances were administered intraperitoneally (i.p.). Solutions were prepared to allow an injection volume of 0.1 mL/100 g body weight.

### 2.3. Y-Maze Apparatus

Spatial memory was evaluated using the Y-maze paradigm. The experimental setup consisted of a Y-shaped structure with three identical arms (47 cm long, 15 cm wide, and 38 cm high) made of wood and covered with adhesive foil to provide a consistent surface texture.

The arms were designated as follows:

- Start arm (S);
- Familiar arm (known arm, K), accessible during both sessions;
- Novel arm (unknown arm, U), blocked during the first session and opened during the second session.

Uniform artificial illumination was ensured across all arms. Noise levels in the experimental room and adjacent areas were minimized. The apparatus was positioned according to fixed visual cues that were maintained constant throughout all of the experiments to prevent spatial bias. Orientation was regularly verified to avoid rotation during sessions.

The animal’s movements within the maze were recorded using a video camera (Logitech, Lausanne, Switzerland) positioned on a support at the lateral end of the start arm (S) and connected to a computer (Lenovo, Morrisville, NC, USA) located in an adjacent room for remote monitoring. This setup allowed continuous observation of the animal and accurate visualization of transitions between maze arms. All video recordings were stored and subsequently analyzed offline to minimize observational bias.

### 2.4. Experimental Procedure

Animals were handled by a single experimenter, who left the room immediately after placing the rat in the maze. Animals were randomly assigned to experimental groups. Behavioral video analyses were performed by an investigator blinded to treatment allocation.

Each experiment consisted of two 5 min sessions in the Y-maze. Drug administration was performed prior to the first session (acquisition phase). To standardize handling and injection timing across groups, all animals received two i.p. injections: the first injection was administered 50 min before Session 1 (donepezil, tacrine, or saline, as applicable), and the second injection was administered 30 min before Session 1 (THP or saline, as applicable). Thus, in groups receiving both THP and a cholinesterase inhibitor, treatments were delivered as two injections separated by 20 min (donepezil/tacrine first, followed by THP). Session 2 (recall) was conducted 24 h after the first maze exposure, without additional drug administration. The administration-to-test intervals were selected to correspond to the expected peak pharmacodynamic activity of the administered compounds [16,17,27,28,29,30].

During the first session, only two arms were accessible: the start arm (S) and the familiar arm (K). The novel arm (U) was closed with a wooden panel identical in texture and appearance to the inner walls and floor of the maze. The position of the familiar and novel arms (left or right) was alternated between animals. Thus, for some animals the right arm was designated as the familiar arm and the left arm as the novel arm, whereas for others the arrangement was reversed. Within each experimental group, half of the animals had the right arm open during the first session and half had the left arm open.

During the second session, all three arms were open, while the designation of the familiar and novel arms was maintained according to the configuration used during the first session.

After each session, the floor of the apparatus was cleaned of animal excretions and wiped with a damp cloth followed by dry wipes in order to minimize olfactory cues for the subsequent animal.

### 2.5. Experimental Design

Two experimental protocols were performed.

(A) Validation experiment (THP dose selection). Three experimental groups were included (*n* = 9 per group), as follows:

(1) C = Control group: 0.1 mL/100 g (i.p.), 0.9% saline;

(2) THP 5 = Trihexyphenidyl 5 mg/kg (i.p.);

(3) THP 10 = Trihexyphenidyl 10 mg/kg (i.p.).

In this experiment, control animals received saline 30 min before Session 1, whereas THP groups received THP 30 min before Session 1.

(B) Intervention experiment under THP challenge (donepezil vs tacrine). Six experimental groups were included (*n* = 9 per group), as follows:

(1) C = Control group: 0.1 mL/100 g (i.p.), 0.9% saline;

(2) THP 10 = Trihexyphenidyl 10 mg/kg (i.p.);

(3) D1 = Donepezil 1 mg/kg (i.p.) + THP 10 mg/kg (i.p.);

(4) D3 = Donepezil 3 mg/kg (i.p.) + THP 10 mg/kg (i.p.);

(5) T3 = Tacrine 3 mg/kg (i.p.) + THP 10 mg/kg (i.p.);

(6) T5 = Tacrine 5 mg/kg (i.p.) + THP 10 mg/kg (i.p.).

In the intervention experiment, donepezil/tacrine (or saline, as applicable) was administered 50 min before Session 1, followed by THP 10 mg/kg (or saline, as applicable) 30 min before Session 1. Control animals received saline injections matched in timing (50 min and 30 min prior to Session 1). The THP 10 group received saline 50 min and THP 30 min prior to Session 1.

Sample size was determined based on previous studies using similar behavioral paradigms reporting comparable variability. Doses were selected based on previous experimental studies demonstrating behavioral effects without inducing motor impairment [16,17,31,32,33,34].

To minimize potential time-related and order-related bias, both drug administration and behavioral testing were performed in an alternating sequence across groups. Specifically, the first animal from each group was injected consecutively (control, THP 10, donepezil 1 mg/kg, donepezil 3 mg/kg, tacrine 3 mg/kg, tacrine 5 mg/kg), followed by the second animal from each group, and so forth, until all animals were treated and tested.

### 2.6. Behavioral Analysis

For analysis, the maze was divided into four zones: the familiar arm (K), the novel arm (U), the intersection zone (I), and the start arm (S).

Acquisition-phase measures (Session 1) included time spent in the start arm (S), time spent in the accessible/familiar arm (K), entries into each arm and total arm entries. Recall-phase measures (24 h) included time spent in the familiar and novel arms (seconds) and the number of entries into the familiar and novel arms.

Based on recall-phase data, the following derived parameters were calculated: the ratio of time spent in the novel arm to time spent in the familiar arm (U/K time ratio), the ratio of the number of entries into the novel arm to the number of entries into the familiar arm (U/K entry ratio), the mean time per entry into the familiar and novel arms, and a discrimination index for both time spent and arm entries, calculated at the individual-animal level as (U − K)/(U + K).

An arm entry was defined as the moment when the animal’s hind paws were fully inside the respective arm. Exit was defined as the moment when the hind paws left the arm.

Higher U/K time and entry ratios were interpreted as indicating relatively greater allocation toward the novel arm during recall. Mean time per entry was interpreted as a supportive index reflecting average dwell time per visit.

### 2.7. Statistical Analysis

Statistical processing of the data was performed using IBM SPSS Statistics for Windows, Version 29.0 (IBM Corp., Armonk, NY, USA). Results were expressed as mean ± standard deviation (SD). Acquisition-phase measures (Session 1) and recall-phase measures (24 h) were analyzed separately by one-way analysis of variance (one-way ANOVA). For recall-phase analysis, the U/K time ratio was designated as the primary outcome. Additional recall-phase variables, including U/K entry ratio, discrimination indices for time and entries, absolute time and entry measures, and mean time per entry, were analyzed as supportive/exploratory outcomes. In the validation experiment, when a significant overall effect was detected, Tukey’s post hoc test was used for multiple comparisons. In the intervention experiment, because the main objective was to compare each treatment group against the THP 10 group, Dunnett’s post hoc test was used for many-to-one comparisons with THP 10 as the reference condition.

Assumptions for parametric testing were assessed using the Shapiro–Wilk test for within-group normality and Levene’s test for homogeneity of variances. Supplementary Table S1 summarizes outcomes showing isolated assumption deviations together with the corresponding sensitivity analyses. In addition, sensitivity analyses using the Kruskal–Wallis test were performed for all outcomes and yielded a concordant overall pattern with the ANOVA-based results.

A value of *p* < 0.05 was considered statistically significant.

## 3. Results

### 3.1. Model Validation (Trihexyphenidyl)

#### 3.1.1. Acquisition-Phase Behavior (Session 1)

Acquisition-phase measures recorded during Session 1, including time spent in the start arm (S), time spent in the accessible/familiar arm (K) and entries into each arm are presented in Table 1.

No statistically significant between-group differences were observed for time spent in the start arm (S) (one-way ANOVA, *p* = 0.847) or in the accessible arm (K) (*p* = 0.399). Similarly, the number of entries into S (*p* = 0.581) and K (*p* = 0.552) did not differ significantly among groups. Total arm entries also showed no significant between-group differences (*p* = 0.447). Together, these findings indicate that the groups were broadly comparable during initial maze exposure and did not exhibit marked differences in acquisition-phase exploratory allocation.

#### 3.1.2. U/K Time Ratio

For recall-phase analysis in the validation experiment, the U/K time ratio was treated as the primary outcome, whereas absolute time and entry measures, U/K entry ratio, discrimination indices and time-per-entry variables were interpreted as supportive/exploratory measures.

**Figure 1:**
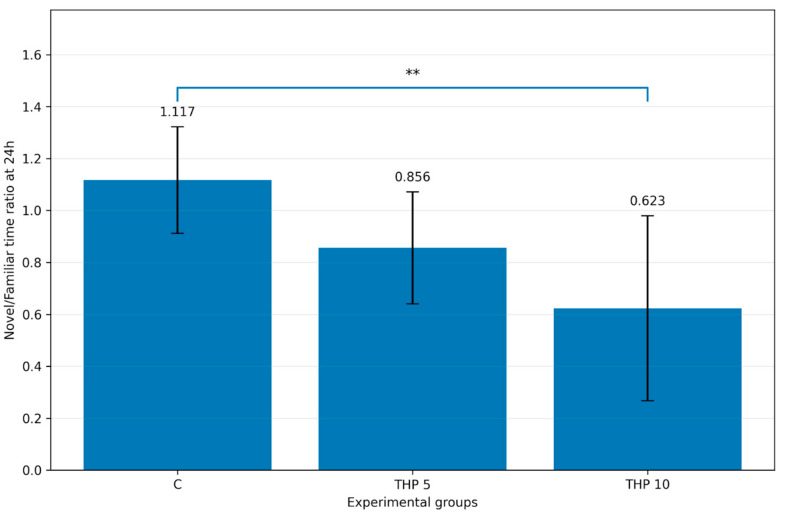
Mean individual U/K time ratio during the second trial (recall session). C = Control group: 0.1 mL/100 g (i.p.), 0.9% saline, THP 5 = Trihexyphenidyl 5 mg/kg (i.p.), THP 10 = Trihexyphenidyl 10 mg/kg (i.p.). Bars represent mean ± SD (mean of individual ratios). The brackets indicate the comparisons and the asterisks the significance levels (** *p* < 0.01).

The U/K time ratio during the recall session is presented in Figure 1.

One-way ANOVA demonstrated a significant treatment effect on the U/K time ratio (F(2,24) = 7.64, *p* = 0.0027). Tukey post hoc analysis revealed that the THP 10 group showed a significantly lower U/K time ratio compared with the control group (*p* = 0.0019), whereas the difference between THP 5 and controls was not significant (*p* = 0.1187). The comparison between THP 10 and THP 5 was also not significant (*p* = 0.1777).

These findings indicate that THP 10 shifted recall-phase exploratory allocation away from the novel arm and toward the familiar arm.

#### 3.1.3. Discrimination Indices

The discrimination index for time, calculated as (U − K)/(U + K), differed significantly among groups (one-way ANOVA: F(2,24) = 9.58, *p* = 0.0009). Mean values were 0.048 ± 0.087 in the control group, −0.090 ± 0.121 in the THP 5 group, and −0.275 ± 0.228 in the THP 10 group. Tukey post hoc analysis showed that THP 10 had a significantly lower time discrimination index than the controls (*p* = 0.0006) and THP 5 (*p* = 0.0497), whereas THP 5 did not differ significantly from the controls (*p* = 0.1718).

The discrimination index for entries, also calculated as (U − K)/(U + K), differed significantly among the groups (one-way ANOVA: F(2,24) = 5.86, *p* = 0.0085). Mean values were 0.100 ± 0.151 in the control group, −0.063 ± 0.239 in the THP 5 group, and −0.282 ± 0.299 in the THP 10 group. Tukey post hoc analysis showed that THP 10 had a significantly lower entry discrimination index than the controls (*p* = 0.0063), whereas no other pairwise comparisons reached statistical significance. These findings further support a THP 10-related shift in exploratory allocation away from the novel arm during recall.

#### 3.1.4. Time Spent in the Familiar and Novel Arms (24 h Recall)

**Figure 2:**
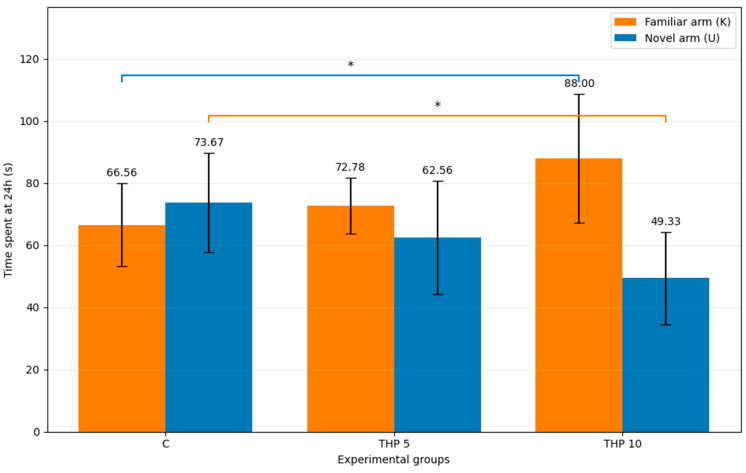
Time spent in novel vs familiar arms (mean ± SD) (seconds) during the second trial (recall session)**.** Bar height represents the mean time spent by the animals in each arm for each experimental group. C = Control group: 0.1 mL/100 g (i.p.), 0.9% saline, THP 5 = Trihexyphenidyl 5 mg/kg (i.p.), THP 10 = Trihexyphenidyl 10 mg/kg (i.p.). The brackets indicate the comparisons and the asterisks the significance levels (* *p* < 0.05).

Mean time spent in the familiar (K) and novel (U) arms during the recall session (24 h) is presented in Table 2 and Figure 2 and was analyzed as a supportive measure.

One-way ANOVA revealed a significant effect of treatment on time spent in the familiar arm (F(2,24) = 4.78, *p* = 0.0179). Tukey post hoc testing indicated that the THP 10 group spent significantly more time in the familiar arm compared with the controls (*p* = 0.0163), whereas THP 5 did not differ significantly from the controls (*p* = 0.6629) or from THP 10 (*p* = 0.1043).

For time spent in the novel arm, a significant treatment effect was also detected (F(2,24) = 4.91, *p* = 0.0163). Tukey post hoc analysis showed that animals treated with THP 10 spent significantly less time in the novel arm compared with the controls (*p* = 0.0122), whereas THP 5 did not differ significantly from the controls (*p* = 0.3420). No other significant pairwise differences were observed.

These findings were directionally consistent with the primary U/K time ratio result and support a THP 10-related shift in recall-phase exploratory allocation away from the novel arm.

#### 3.1.5. Number of Entries into Familiar and Novel Arms

**Figure 3:**
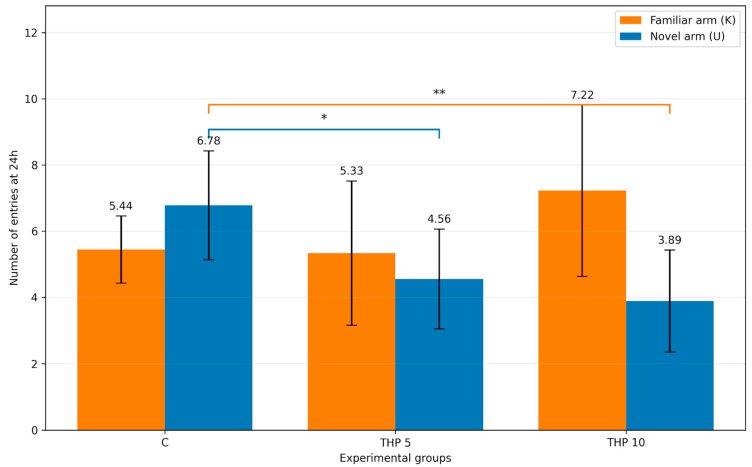
Number of entries into novel vs familiar arms (mean ± SD) during the second trial (recall session). The height of the columns represents the mean number of entries into each arm for each experimental group. C = Control group: 0.1 mL/100 g (i.p.), 0.9% saline, THP 5 = Trihexyphenidyl 5 mg/kg (i.p.), THP 10 = Trihexyphenidyl 10 mg/kg (i.p.). The brackets indicate the comparisons and the asterisks the significance levels (* *p* < 0.05, ** *p* < 0.01).

The number of entries into the familiar and novel arms at 24 h is summarized in Table 3 and Figure 3.

One-way ANOVA did not detect a significant treatment effect for entries into the familiar arm (F(2,24) = 2.43, *p* = 0.109). In contrast, a significant treatment effect was observed for entries into the novel arm (F(2,24) = 8.42, *p* = 0.0017). Tukey post hoc testing showed that both THP 5 and THP 10 groups made significantly fewer entries into the novel arm compared with controls (*p* = 0.0159 and *p* = 0.0018, respectively). No significant difference was detected between THP 10 and THP 5 (*p* = 0.6427).

Total entries (K + U) did not differ significantly among groups (F(2,24) = 2.05, *p* = 0.150).

#### 3.1.6. U/K Entry Ratio

**Figure 4:**
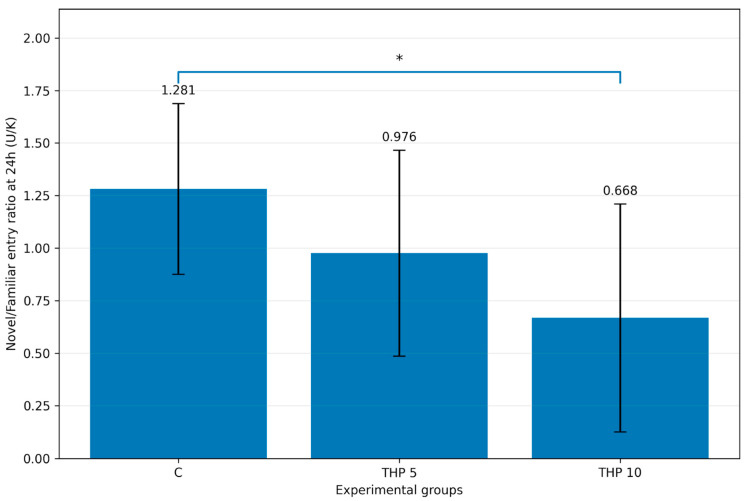
Mean U/K entry ratio during the second trial (recall session). C = Control group: 0.1 mL/100 g (i.p.), 0.9% saline; THP 5 = trihexyphenidyl 5 mg/kg (i.p.); THP 10 = trihexyphenidyl 10 mg/kg (i.p.). Bars represent mean ± SD (mean of individual ratios). The brackets indicate the comparisons and the asterisks the significance levels (* *p* < 0.05).

Mean individual U/K entry ratio decreased across THP doses (C: 1.281 ± 0.406, THP 5: 0.976 ± 0.490, THP 10: 0.668 ± 0.542), as illustrated in Figure 4. One-way ANOVA indicated a significant treatment effect (F(2,24) = 3.64, *p* = 0.0416). Tukey post hoc analysis showed that THP 10 had a significantly lower U/K entry ratio compared with controls (*p* = 0.0324), whereas THP 5 did not differ significantly from controls (*p* = 0.3855). No other pairwise differences were significant.

These findings indicate that THP 10 shifted exploratory allocation away from the novel arm, an effect that was also captured by the ratio-based entry parameter.

#### 3.1.7. Mean Time per Entry

**Figure 5:**
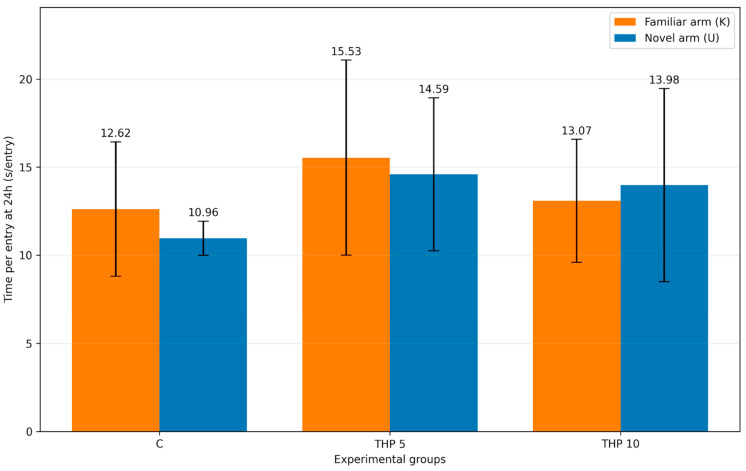
Mean time per entry into the familiar and novel arms (mean ± SD) during the second trial (recall session). C = Control group: 0.1 mL/100 g (i.p.), 0.9% saline; THP 5 = trihexyphenidyl 5 mg/kg (i.p.); THP 10 = trihexyphenidyl 10 mg/kg (i.p.).

Mean time per entry into the familiar and novel arms is presented in Table 4 and Figure 5.

To account for potential differences in visit frequency, time per entry was calculated for each animal by dividing the time spent in each arm by the number of entries into the respective arm.

For familiar-arm time per entry (K), no significant treatment effect was detected (one-way ANOVA: F(2,24) = 1.16, *p* = 0.332). Likewise, novel-arm time per entry (U) did not differ significantly between groups (F(2,24) = 2.05, *p* = 0.151). Tukey post hoc testing did not reveal any significant pairwise differences for either parameter.

These results suggest that group effects observed in time- and entry-based measures primarily reflect differences in arm selection rather than prolonged occupancy per visit.

### 3.2. Effects of Donepezil and Tacrine Under THP-Induced Cholinergic Impairment

#### 3.2.1. Acquisition-Phase Behavior (Session 1)

Behavioral measures recorded during the acquisition session are summarized in Table 5. No statistically significant between-group differences were observed for time spent in the start arm (S) (one-way ANOVA, *p* = 0.912) or in the accessible arm (K) (*p* = 0.916). Similarly, the number of entries into S (*p* = 0.901) and K (*p* = 0.672) did not differ significantly among groups. Total arm entries also showed no significant between-group differences (*p* = 0.186). Together, these findings indicate that the groups were broadly comparable during initial maze exposure and did not exhibit marked differences in acquisition-phase exploratory behavior.

#### 3.2.2. U/K Time Ratio

For recall-phase analysis in the intervention experiment, the U/K time ratio was treated as the primary outcome, whereas the remaining recall-phase variables were interpreted as supportive/exploratory measures.

**Figure 6:**
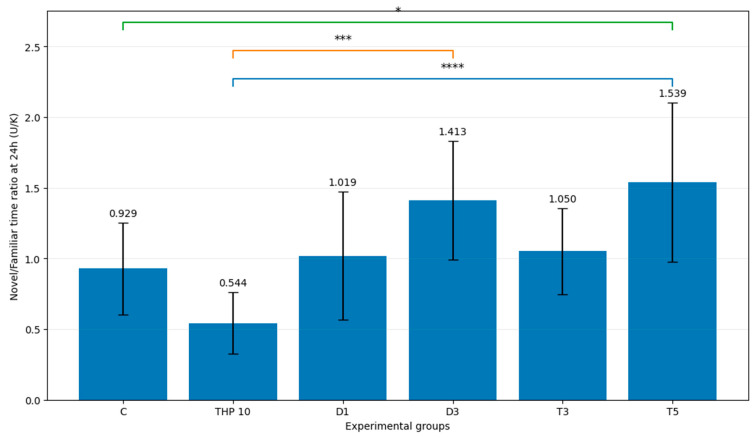
Mean individual U/K time ratio during the second trial (recall session). C = Control group: 0.1 mL/100 g (i.p.), 0.9% saline, THP 10 = Trihexyphenidyl 10 mg/kg (i.p.), D1 = Donepezil 1 mg/kg (i.p.) + THP 10 mg/kg (i.p.), D3 = Donepezil 3 mg/kg (i.p.) + THP 10 mg/kg (i.p.), T3 = Tacrine 3 mg/kg (i.p.) + THP 10 mg/kg (i.p.), T5 = Tacrine 5 mg/kg + THP 10 mg/kg (i.p.). Bars represent mean ± SD (mean of individual ratios). The brackets indicate the comparisons and the asterisks the significance levels (* *p* < 0.05, *** *p* < 0.001, **** *p* < 0.0001). Colored lines/brackets are used only to visually distinguish separate pairwise comparisons.

The U/K time ratio during the recall session is presented in Figure 6.

One-way ANOVA demonstrated a significant treatment effect on the U/K time ratio (F(5,48) = 7.30, *p* < 0.001). In many-to-one post hoc comparisons using Dunnett’s test with THP 10 as the reference group, the D3 (*p* = 0.0001), T3 (*p* = 0.0379), and T5 (*p* < 0.0001) groups showed significantly higher U/K time ratio values than THP 10, whereas the differences for the control group (*p* = 0.1608) and D1 (*p* = 0.0566) were not significant.

These results indicate that, relative to THP-induced impairment, donepezil at 3 mg/kg and tacrine at 3 and 5 mg/kg were associated with a shift in exploratory allocation toward the novel arm, as reflected by an increased U/K time ratio.

#### 3.2.3. Discrimination Indices

The discrimination index for time, calculated as (U − K)/(U + K), differed significantly among groups (one-way ANOVA: F(5,48) = 8.90, *p* = 4.96 × 10^−6^). Mean values were −0.065 ± 0.192 in the control group, −0.320 ± 0.201 in the THP 10 group, −0.033 ± 0.219 in the D1 group, 0.149 ± 0.143 in the D3 group, 0.006 ± 0.143 in the T3 group, and 0.179 ± 0.172 in the T5 group. In Dunnett’s post hoc comparisons against THP 10, the time discrimination index was significantly higher in the control (*p* = 0.0182), D1 (*p* = 0.0066), D3 (*p* < 0.0001), T3 (*p* = 0.0017), and T5 (*p* < 0.0001) groups.

The discrimination index for entries also differed significantly among groups (one-way ANOVA: F(5,48) = 8.37, *p* = 9.52 × 10^−6^). Mean values were 0.034 ± 0.116 in the control group, −0.238 ± 0.196 in the THP 10 group, −0.066 ± 0.143 in the D1 group, 0.118 ± 0.139 in the D3 group, 0.135 ± 0.165 in the T3 group, and 0.135 ± 0.151 in the T5 group. In Dunnett’s post hoc comparisons against THP 10, the entry discrimination index was significantly higher in the control (*p* = 0.0022), D3 (*p* = 0.0001), T3 (*p* < 0.0001), and T5 (*p* < 0.0001) groups, whereas the comparison with D1 was not significant (*p* = 0.0852).

Together, these findings support a treatment-related shift in exploratory allocation toward the novel arm relative to THP 10 alone.

#### 3.2.4. Time Spent in the Familiar and Novel Arms (24 h Recall)

**Figure 7:**
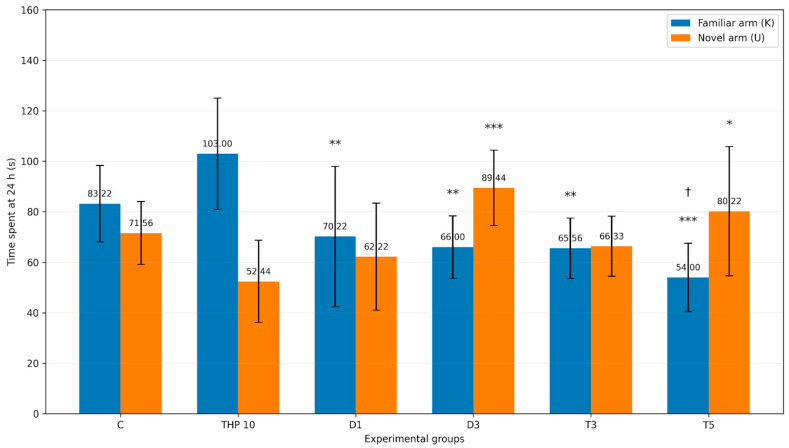
Time spent in novel vs familiar arms (mean ± SD) (seconds) during the second trial (recall session)**.** Bar height represents the mean time spent by the animals in each arm for each experimental group. C = Control group: 0.1 mL/100 g (i.p.), 0.9% saline, THP 10 = Trihexyphenidyl 10 mg/kg (i.p.), D1 = Donepezil 1 mg/kg (i.p.) + THP 10 mg/kg (i.p.), D3 = Donepezil 3 mg/kg (i.p.) + THP 10 mg/kg (i.p.), T3 = Tacrine 3 mg/kg (i.p.) + THP 10 mg/kg (i.p.), T5 = Tacrine 5 mg/kg + THP 10 mg/kg (i.p.). Symbols above the bars indicate significance levels versus the THP 10 group within the same arm (* *p* < 0.05, ** *p* < 0.01, *** *p* < 0.001). † indicates *p* < 0.05 versus the control group within the same arm.

Mean time spent in the familiar (K) and novel (U) arms during the recall session (24 h) is presented in Table 6 and Figure 7.

Compared with the THP 10 group, treatment with donepezil 3 mg/kg and tacrine 5 mg/kg was associated with a redistribution of exploration toward the novel arm, reflected by less time spent in the familiar arm and more time spent in the novel arm.

Regarding the familiar arm (K), one-way ANOVA revealed a significant effect of treatment on time spent in the familiar arm (F(5,48) = 7.16, *p* = 4.51 × 10^−5^). In Dunnett’s post hoc comparisons against THP 10, time spent in the familiar arm was significantly lower in the D1 (*p* = 0.0033), D3 (*p* = 0.0008), T3 (*p* = 0.0007), and T5 (*p* < 0.0001) groups, whereas the difference for the control group was not significant (*p* = 0.1283).

Regarding the novel arm (U), a significant treatment effect was also detected (F(5,48) = 4.94, *p* = 0.0010). Dunnett’s post hoc analysis showed that the D3 (*p* = 0.0003) and T5 (*p* = 0.0077) groups spent significantly more time in the novel arm than THP 10, whereas the differences for the control group (*p* = 0.1029), D1 (*p* = 0.6588), and T3 (*p* = 0.3368) were not significant.

#### 3.2.5. Number of Entries into Familiar and Novel Arms

**Figure 8:**
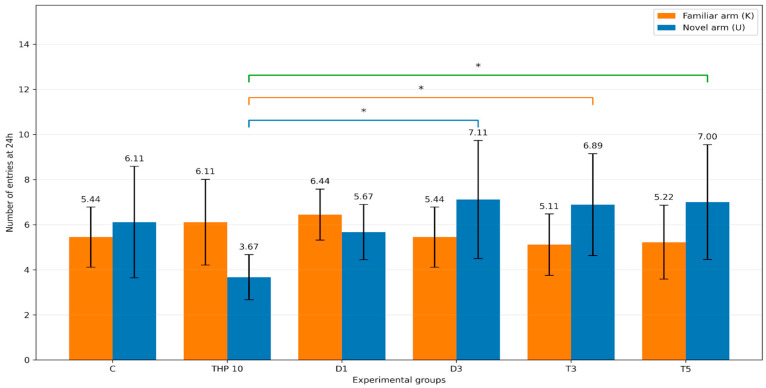
Number of entries into the novel and familiar arms (mean ± SD) during the second trial (recall session). C = Control group: 0.1 mL/100 g (i.p.), 0.9% saline, THP 10 = Trihexyphenidyl 10 mg/kg (i.p.), D1 = Donepezil 1 mg/kg (i.p.) + THP 10 mg/kg (i.p.), D3 = Donepezil 3 mg/kg (i.p.) + THP 10 mg/kg (i.p.), T3 = Tacrine 3 mg/kg (i.p.) + THP 10 mg/kg (i.p.), T5 = Tacrine 5 mg/kg + THP 10 mg/kg (i.p.). The brackets indicate the comparisons and the asterisks the significance levels (* *p* < 0.05). Colored lines/brackets are used only to visually distinguish separate pairwise comparisons.

The number of entries into the familiar and novel arms at 24 h is summarized in Table 7 and Figure 8.

Regarding familiar-arm (K) entries, one-way ANOVA did not detect a significant treatment effect (F(5,48) = 1.16, *p* = 0.341).

Regarding novel-arm (U) entries, one-way ANOVA revealed a significant effect of treatment (F(5,48) = 3.41, *p* = 0.010). In Dunnett’s post hoc comparisons against THP 10, the D3 (*p* = 0.0055), T3 (*p* = 0.0102), and T5 (*p* = 0.0075) groups made significantly more entries into the novel arm, whereas the differences for the control group (*p* = 0.0726) and D1 (*p* = 0.1845) were not significant.

These findings indicate that THP-induced reduction in novel-arm exploration was attenuated in the D3, T3, and T5 groups.

#### 3.2.6. U/K Entry Ratio

**Figure 9:**
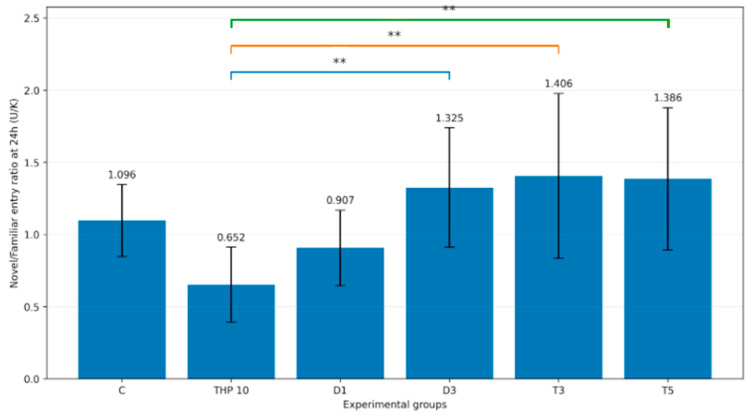
Mean U/K entry ratio during the second trial (recall session). C = Control group: 0.1 mL/100 g (i.p.), 0.9% saline, THP 10 = Trihexyphenidyl 10 mg/kg (i.p.), D1 = Donepezil 1 mg/kg (i.p.) + THP 10 mg/kg (i.p.), D3 = Donepezil 3 mg/kg (i.p.) + THP 10 mg/kg (i.p.), T3 = Tacrine 3 mg/kg (i.p.) + THP 10 mg/kg (i.p.), T5 = Tacrine 5 mg/kg + THP 10 mg/kg (i.p.). Bars represent mean ± SD (mean of individual ratios). The brackets indicate the comparisons and the asterisks the significance levels (** *p* < 0.01). Colored lines/brackets are used only to visually distinguish separate pairwise comparisons.

One-way ANOVA demonstrated a significant treatment effect on the U/K entry ratio (F(5,48) = 5.28, *p* = 0.0006). Mean values for each group are shown in Figure 9. In Dunnett’s post hoc comparisons using THP 10 as the reference group, the D3 (*p* = 0.0034), T3 (*p* = 0.0009), and T5 (*p* = 0.0012) groups exhibited significantly higher U/K entry ratio values than THP 10, whereas the comparisons with the control group (*p* = 0.0825) and D1 (*p* = 0.5179) were not significant.

Thus, ratio-based entry measures supported the shift in arm-selection dynamics observed in the treated groups relative to THP 10 alone.

#### 3.2.7. Mean Time per Entry

**Figure 10:**
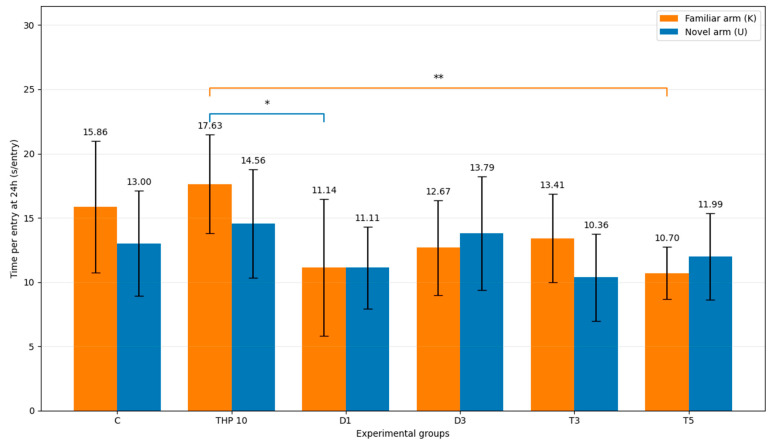
Mean time per entry into the familiar and novel arms (mean ± SD) (seconds) during the second trial (recall session). C = Control group: 0.1 mL/100 g (i.p.), 0.9% saline, THP 10 = Trihexyphenidyl 10 mg/kg (i.p.), D1 = Donepezil 1 mg/kg (i.p.) + THP 10 mg/kg (i.p.), D3 = Donepezil 3 mg/kg (i.p.) + THP 10 mg/kg (i.p.), T3 = Tacrine 3 mg/kg (i.p.) + THP 10 mg/kg (i.p.), T5 = Tacrine 5 mg/kg + THP 10 mg/kg (i.p.). Bars represent mean ± SD. The brackets indicate the comparisons and the asterisks the significance levels (* *p* < 0.05, ** *p* < 0.01).

Mean time per entry into the familiar and novel arms is presented in Table 8 and Figure 10.

Regarding familiar-arm (K) time per entry, one-way ANOVA indicated a significant treatment effect (F(5,48) = 4.00, *p* = 0.0041). In Dunnett’s post hoc comparisons against THP 10, familiar-arm time per entry was significantly lower in the D1 (*p* = 0.0063) and T5 (*p* = 0.0032) groups, whereas the differences for the control group (*p* = 0.8232), D3 (*p* = 0.0512), and T3 (*p* = 0.1223) were not significant.

Regarding novel-arm (U) time per entry, no significant treatment effect was detected (F(5,48) = 1.60, *p* = 0.177), and Dunnett’s post hoc comparisons did not identify significant differences versus THP 10.

## 4. Discussion

In this study, we first characterized a pharmacological impairment paradigm using trihexyphenidyl (THP) and then, in THP-challenged animals, compared the effects of selective acetylcholinesterase (AChE) inhibition (donepezil) versus dual AChE/butyrylcholinesterase (BuChE) inhibition (tacrine) on recall-phase exploratory allocation in a two-trial Y-maze paradigm with a 24 h interval.

In the validation experiment, THP at 10 mg/kg produced a consistent redistribution of exploration during the 24 h recall session, with increased time spent in the familiar arm and reduced time spent in the novel arm, accompanied by a significant decrease in the U/K time ratio. This convergence of absolute time measures with a ratio-based metric supports the interpretation that the higher THP dose produced a robust recall-phase phenotype under the present conditions. Centrally acting antimuscarinic compounds are widely used to induce transient cholinergic disruption and reproducible deficits in rodent cognition-related behavioral paradigms, supporting their use as pharmacological challenge models. Moreover, THP-induced impairment has been applied as a challenge paradigm for assessing cognition-directed interventions in rodents [16,35,36].

In contrast, THP 5 mg/kg did not differ significantly from controls for time spent in either arm or for the U/K time ratio, indicating that the impairment phenotype was most robust at 10 mg/kg in this paradigm. Entry-based measures further supported this pattern. While familiar-arm entries did not show a significant treatment effect, both THP 5 and THP 10 reduced novel-arm entries relative to controls, and total entries did not differ significantly among groups. The combination of reduced novel-arm engagement in the absence of a clear reduction in overall entry counts argues against a simple global suppression of exploratory activity and instead supports a shift in allocation away from novelty under muscarinic antagonism. Consistent with this interpretation, THP has been reported to reduce spontaneous alternation performance in an automated Y-maze toward near-chance levels, highlighting the sensitivity of arm-selection metrics to muscarinic blockade [21].

Importantly, time-per-entry analysis did not reveal significant differences between validation groups, suggesting that the THP-related effects on time and entries are more consistent with altered arm selection/allocation than with prolonged occupancy per visit. This interpretation is consistent with Y-maze approaches that emphasize sequential choice structure (i.e., patterns derived from successive arm selections) as readouts of working memory and executive function [37].

Taken together, these results support using THP 10 mg/kg as a cholinergic challenge dose, which produced a robust shift in recall-phase exploratory allocation in this 24 h Y-maze paradigm. In our dataset, the U/K time ratio was particularly informative because it captured competitive allocation between arms while partially reducing inter-individual variability in overall exploration. The remaining measures were directionally concordant with this pattern.

In the full six-group experiment (control, THP 10, donepezil 1 and 3 mg/kg, tacrine 3 and 5 mg/kg), the primary pattern again emerged as a redistribution of exploration between arms rather than a uniform change in overall activity. For time spent in the familiar arm, THP 10 showed increased persistence relative to multiple treated groups, whereas tacrine 5 mg/kg produced a marked reduction in familiar-arm time (including a significant reduction relative to controls). For the novel arm, donepezil 3 mg/kg and tacrine 5 mg/kg increased novel-arm time relative to THP 10, with donepezil 3 mg/kg also exceeding donepezil 1 mg/kg. Overall, under THP-induced impairment, higher-dose donepezil—and especially higher-dose tacrine—shifted recall-phase allocation toward the novel option.

The ratio-based outcomes were particularly informative. The U/K time ratio showed a clear treatment effect, with significantly higher values in D3, T3, and T5 relative to THP 10. The U/K entry ratio showed a concordant pattern, with significantly higher values in D3, T3, and T5 relative to THP 10. The concordance between time-ratio and entry-ratio measures strengthens the interpretation that treatment effects represent a consistent shift in competitive allocation between arms rather than an artifact of a single behavioral metric.

Entry analyses in the six-group experiment provide additional information. Familiar-arm entries did not differ significantly across groups, whereas novel-arm entries showed a significant treatment effect: THP 10 made fewer novel-arm entries compared with donepezil 3 mg/kg and both tacrine groups. This pattern indicates that THP-related impairment includes reduced engagement with the novel option and that treatment effects (particularly tacrine and high-dose donepezil) shift novelty-directed choices relative to THP 10 alone. Notably, tacrine 3 mg/kg was more evident in entry-based outcomes than in time-ratio outcomes, suggesting that at this dose the behavioral impact may primarily reflect the frequency of selecting the novel arm rather than a large change in dwell time once the arm is entered.

Time-per-entry analysis was included to test whether group differences reflect prolonged visits (dwell time per entry) rather than arm choice. In this six-group experiment, familiar-arm time per entry showed a significant effect, with THP 10 higher than donepezil 1 mg/kg and tacrine 5 mg/kg, while novel-arm time per entry did not differ significantly among groups. This profile is consistent with THP increasing persistence once the familiar arm is chosen, while some treatments reduce this persistence. However, because time-per-entry is a derived variable (time divided by entries) and can be sensitive to variability in entry counts, it is best interpreted as supportive evidence reinforcing the primary conclusion: across the dataset, treatment effects are driven predominantly by arm allocation/selection rather than by generalized prolongation of occupancy.

Donepezil and tacrine showed partially overlapping but distinct profiles under THP challenge. Donepezil exhibited its clearest effects at 3 mg/kg, increasing novel-arm time and the U/K time ratio relative to THP 10, with concordant improvements in novel-arm entries and the U/K entry ratio; the 1 mg/kg dose showed more limited modulation. Tacrine displayed a broader dose-dependent signature: at 3 mg/kg it was more apparent in entry-based measures than in time-ratio outcomes, suggesting an effect primarily on the frequency of selecting the novel arm, whereas at 5 mg/kg it produced the most robust redistribution of time allocation, reducing familiar-arm time (including relative to controls) and increasing the U/K time ratio relative to both THP 10 and controls, alongside improved entry-based indices. Mean time-per-entry did not differ in the novel arm, while familiar-arm time-per-entry was elevated by THP 10 and reduced by tacrine 5 mg/kg, supporting that the dominant effects involve arm-allocation dynamics rather than generalized changes in visit duration.

However, tacrine’s broader dose–response profile should not be interpreted only in terms of dual AChE/BuChE inhibition [24,25]. An equally plausible interpretation is that the stronger effects observed at 5 mg/kg reflect tacrine’s broader pharmacology beyond cholinesterase inhibition, including reported interactions with muscarinic and nicotinic receptor systems [38,39], monoamine oxidases [40], and potassium channels [41]. In this context, the apparent overshoot-like pattern observed in some outcomes relative to healthy controls may indicate a qualitatively distinct behavioral state rather than simple restoration to baseline. Such effects could involve altered arousal, novelty-seeking, or other composite pharmacodynamic influences under THP challenge.

These findings contribute to refining pharmacological Y-maze paradigms for evaluating cholinergic modulation in preclinical models of cognitive impairment.

### Limitations

Several limitations should be considered. First, Y-maze exploration can be influenced by non-mnestic factors such as anxiety, arousal, and novelty seeking; although entry counts and time-per-entry patterns do not suggest major global suppression of exploration, complementary behavioral assays (e.g., open-field or elevated plus maze) would help disentangle mnemonic from affective contributors. Second, the intervention experiment did not include donepezil-alone or tacrine-alone control groups; therefore, treatment-related changes cannot be attributed specifically to reversal of THP-induced cholinergic impairment, and values exceeding those of healthy controls should not be interpreted as simple restoration to baseline. Third, the study relied on acute pharmacological manipulations, and dose- and timing-dependent pharmacokinetics may have influenced effect magnitude. In addition, the present study did not include biochemical or receptor-level validation of cholinergic disruption, so interpretation remains based on behavioral pharmacology rather than direct mechanistic confirmation. Moreover, the acute THP challenge used here does not reproduce the chronic and multifactorial pathophysiology of Alzheimer’s disease, but rather models an acute pharmacologically induced impairment state that may capture selected cholinergic components relevant to cognitive dysfunction in Alzheimer’s disease. Finally, because ratio-based measures can be sensitive to denominator variability, inference should remain anchored to convergent patterns across endpoints, with the U/K time ratio defined as the primary recall-phase outcome and the remaining measures interpreted as supportive.

## 5. Conclusions

In this study, trihexyphenidyl (THP) at 10 mg/kg produced a robust recall-phase exploratory phenotype in a 24 h two-trial Y-maze paradigm, characterized by redistribution of exploration toward the familiar arm. Under this challenge, cholinesterase inhibitors modulated exploratory allocation, with donepezil showing clearer effects at 3 mg/kg and tacrine producing a broader dose-dependent profile, most pronounced at 5 mg/kg. The U/K time ratio was the most informative recall-phase measure, while the remaining ratio-based and entry-based outcomes were directionally concordant with this pattern. These findings support the potential utility of trihexyphenidyl-based paradigms in preclinical research on cholinergic dysfunction.

## Figures and Tables

**Table 1:** Behavioral measures recorded during the acquisition session (mean ± SD).

Arm	C Mean ± SD (s)	THP 5 Mean ± SD (s)	THP 10 Mean ± SD (s)
Time in S arm (s)	121.44 ± 16.54	130.67 ± 45.30	127.33 ± 34.51
Time in K arm (s)	112.44 ± 31.62	110.33 ± 33.68	128.89 ± 28.06
Entries into S arm	7.56 ± 2.83	6.89 ± 1.96	8.22 ± 3.11
Entries into K arm	8.67 ± 2.74	7.78 ± 2.68	7.33 ± 2.40

**Table 2:** Time spent in the novel arm (U) and familiar arm (K) at 24 h.

Arm	C Mean ± SD (s)	THP 5 Mean ± SD (s)	THP 10 Mean ± SD (s)
Novel arm (U)	73.67 ± 16.02	62.56 ± 18.28	49.33 ± 14.98
Familiar arm (K)	66.56 ± 13.32	72.78 ± 8.96	88.00 ± 20.74

**Table 3:** Number of entries in the novel arm (U) and familiar arm (K) at 24 h.

Arm	C Mean ± SD	THP 5 Mean ± SD	THP 10 Mean ± SD
Novel arm (U)	6.78 ± 1.64	4.56 ± 1.51	3.89 ± 1.54
Familiar arm (K)	5.44 ± 1.0	5.33 ± 2.18	7.22 ± 2.59

**Table 4:** Mean time per entry in the novel arm (U) and familiar arm (K) at 24 h.

Arm	C Mean ± SD (s)	THP 5 Mean ± SD (s)	THP 10 Mean ± SD (s)
Novel arm (U)	10.96 ± 0.97	14.59 ± 4.34	13.98 ± 5.48
Familiar arm (K)	12.62 ± 3.82	15.53 ± 5.54	13.07 ± 3.49

**Table 5:** Behavioral measures recorded during the acquisition session (mean ± SD).

Arm	C Mean ± SD (s)	THP 10 Mean ± SD (s)	D1 Mean ± SD (s)	D3 Mean ± SD (s)	T3 Mean ± SD (s)	T5 Mean ± SD (s)
Time in S arm (s)	116.00 ± 32.83	122.33 ± 38.96	119.56 ± 43.74	135.56 ± 46.45	111.44 ± 56.76	125.44 ± 52.42
Time in K arm (s)	122.11 ± 28.89	110.11 ± 37.49	104.22 ± 44.23	109.56 ± 46.40	123.00 ± 50.71	110.78 ± 40.81
Entries into S arm	7.67 ± 2.24	8.78 ± 2.28	8.89 ± 2.80	8.78 ± 2.86	7.89 ± 2.52	8.44 ± 3.57
Entries into K arm	8.33 ± 2.96	9.22 ± 2.77	8.22 ± 2.17	9.11 ± 3.10	6.89 ± 4.01	8.00 ± 3.74

**Table 6:** Time spent in the novel arm (U) and familiar arm (K) at 24 h.

Arm	C Mean ± SD (s)	THP 10 Mean ± SD (s)	D1 Mean ± SD (s)	D3 Mean ± SD (s)	T3 Mean ± SD (s)	T5 Mean ± SD (s)
Novel arm (U)	71.56 ± 12.46	52.44 ± 16.33	62.22 ± 21.22	89.44 ± 14.92	66.33 ± 11.91	80.22 ± 25.61
Familiar arm (K)	83.22 ± 15.14	103.00 ± 22.03	70.22 ± 27.77	66.00 ± 12.39	65.56 ± 11.97	54.00 ± 13.55

**Table 7:** Number of entries in the novel arm (U) and familiar arm (K) at 24 h.

Arm	C Mean ± SD (Entries)	THP 10 Mean ± SD (Entries)	D1 Mean ± SD (Entries)	D3 Mean ± SD (Entries)	T3 Mean ± SD (Entries)	T5 Mean ± SD (Entries)
Novel arm (U)	6.11 ± 2.47	3.67 ± 1.00	5.67 ± 1.22	7.11 ± 2.62	6.89 ± 2.26	7.00 ± 2.55
Familiar arm (K)	5.44 ± 1.33	6.11 ± 1.90	6.44 ± 1.13	5.44 ± 1.33	5.11 ± 1.36	5.22 ± 1.64

**Table 8:** Mean time per entry in the novel arm (U) and familiar arm (K) at 24 h.

Arm	C Mean ± SD (s)	THP 10 Mean ± SD (s)	D1 Mean ± SD (s)	D3 Mean ± SD (s)	T3 Mean ± SD (s)	T5 Mean ± SD (s)
Novel arm (U)	13.00 ± 4.09	14.56 ± 4.22	11.11 ± 3.17	13.79 ± 4.43	10.36 ± 3.40	11.99 ± 3.36
Familiar arm (K)	15.86 ± 5.17	17.63 ± 3.9	11.14 ± 5.54	12.67 ± 3.75	13.41 ± 3.49	10.70 ± 2.02

## Data Availability

The data generated and analyzed during the current study are available from the author on request.

## Supplementary Materials

The following supporting information can be downloaded at: https://www.mdpi.com/article/10.3390/biomedicines14040938/s1. Table S1: Assumption checks and sensitivity analyses for outcomes showing isolated deviations from parametric assumptions.

## Abbreviations

The following abbreviations are used in this manuscript:

AD	Alzheimer’s disease
THP	trihexyphenidyl
AChE	acetylcholinesterase
BuChE	butyrylcholinesterase
i.p.	intraperitoneal
ARRIVE	Animal Research: Reporting of In Vivo Experiments
SD	standard deviation
ANOVA	analysis of variance
