# Supplementary material for: Differential Effects of Donepezil and Tacrine on Recall-Phase Exploration in a Trihexyphenidyl-Induced Cholinergic Impairment Y-Maze Model
Source: Biomedicines. 2026 Apr 20;14(4):938. doi: 10.3390/biomedicines14040938 (PMC13114100; doi:10.3390/biomedicines14040938)
Supplement: Supplementary file 1 [file biomedicines-14-00938-s001.zip › biomedicines-4236953-supplementary.pdf]

Table S1. Assumption checks and sensitivity analyses for outcomes showing isolated deviations from parametric assumptions.

Experiment	Outcome	Assumption issue	Shapiro–Wilk / Levene result	Sensitivity analysis	Conclusion
**Validation**	U/K time ratio	Isolated normality deviation in THP 10	Shapiro–Wilk p = 0.0326	Kruskal–Wallis p = 0.0040	Concordant with ANOVA
**Validation**	U/K entry ratio	Isolated normality deviation in THP 10	Shapiro–Wilk p = 0.0044	Kruskal–Wallis p = 0.0088	Concordant with ANOVA
**Intervention**	Time spent in novel arm (U)	Isolated normality deviation in control group	Shapiro–Wilk p = 0.0173	Kruskal–Wallis p = 0.0027	Concordant with ANOVA
**Intervention**	Novel-arm entries (U)	Isolated normality deviation in D3	Shapiro–Wilk p = 0.0132	Kruskal–Wallis p = 0.0056	Concordant with ANOVA
**Intervention**	U/K entry ratio	Isolated normality deviations in T3 and T5	Shapiro–Wilk p = 0.0329 (T3); p = 0.0397 (T5)	Kruskal–Wallis p = 0.0004	Concordant with ANOVA
